# MRI in Chronic Aortic Dissection: A Systematic Review and Future Directions

**DOI:** 10.3389/fcvm.2015.00005

**Published:** 2015-02-19

**Authors:** Andrew G. Sherrah, Stuart M. Grieve, Richmond W. Jeremy, Paul G. Bannon, Michael P. Vallely, Rajesh Puranik

**Affiliations:** ^1^Sydney Medical School, University of Sydney, Sydney, NSW, Australia; ^2^The Baird Institute, Royal Prince Alfred Hospital, Sydney, NSW, Australia; ^3^Department of Radiology, Royal Prince Alfred Hospital, Sydney, NSW, Australia; ^4^Charles Perkins Centre, University of Sydney, Sydney, NSW, Australia; ^5^Heart Research Institute, University of Sydney, Sydney, NSW, Australia; ^6^Australian School of Advanced Medicine, Macquarie University, Sydney, NSW, Australia; ^7^Cardiovascular Magnetic Resonance Sydney, Sydney, NSW, Australia

**Keywords:** chronic aortic dissection, aortic type B dissection, aortic type A dissection, magnetic resonance imaging, follow-up

## Abstract

The acute event of thoracic aortic dissection carries with it high mortality and morbidity. Despite optimal initial surgical or medical management strategies, the risk of further complications in the long-term, including aneurysmal dilatation and false lumen (FL) expansion, are not insignificant. Adequate follow-up of such conditions requires dedicated imaging where relevant prognostic indicators are accurately assessed. We perform a systematic review of the literature and report the current evidence for the use of magnetic resonance imaging (MRI) in assessment of chronic aortic dissection. We then make a comparison with traditional imaging modalities including computed tomography and echocardiography. We discuss new ways in which MRI may extend existing aortic assessment, including identification of blood-flow dynamics within the TL and FL using phase-contrast imaging.

## Introduction

Aortic dissection is a catastrophic complication of aortic wall disease associated with high mortality and morbidity. The underlying process of the aortic wall disruption is most commonly secondary to atherosclerotic disease (especially with older age) or a known connective tissue disease [such as Marfan syndrome (MFS); thoracic aortic aneurysm and dissection syndrome (TAAD); or bicuspid aortic valve (BAV)]. Using data from the International Registry of Acute Aortic Dissection (IRAD), aortic dissection is more common in men, with mean age of 63 years and an incidence of up to 0.8% at autopsy (1–4). According to the Stanford classification, type A aortic dissections involve the proximal/ascending aorta (and may extend distally) while type B aortic dissections involve the descending thoracic aorta without any proximal extension (5, 6). The aims of surgical intervention in type A dissection are the prevention of aortic rupture, severe aortic valve regurgitation, coronary artery dissection, and cardiac tamponade (2). Compared with type B dissection, type A confers a higher risk of neurological complications, including stroke. For type B, surgical or endovascular intervention is typically reserved only for those with clinical compromise or who are inadequately managed with medical therapy alone (2, 3).

It is well recognized that persisting or chronic aortic dissection (>2 weeks after initial intimal injury) is a risk factor for further aortic dilatation and dissection extension (7–9). In these cases, intervention may be required to prevent progressive aortic dilatation (1). Re-operation rates in type A dissection for areas of aneurysmal dilatation or persisting dissection range from 10 to 20% in the first 10 years following initial surgery (10).

A key issue regarding the management of chronic aortic dissections is a progressive increase in the size of the false lumen (FL). The reported incidence of partial or complete distal aortic FL patency in type A dissection patients is significant, ranging between 31 and 89% (9). For type B dissection patients who have been medically managed, a persisting FL in the chronic phase correlates with an increased risk of aortic enlargement (11). Hence, regardless of the initial management in aortic dissection, careful follow-up with appropriate imaging is mandated (12). Several international guidelines recommend the close follow-up of these patients; however multiple imaging modalities are currently used without a clear consensus of the gold standard (2–5). This “gap” in the evidence is specifically noted in the current European Society of Cardiology guidelines on aortic disease management (4).

**Figure 1:**
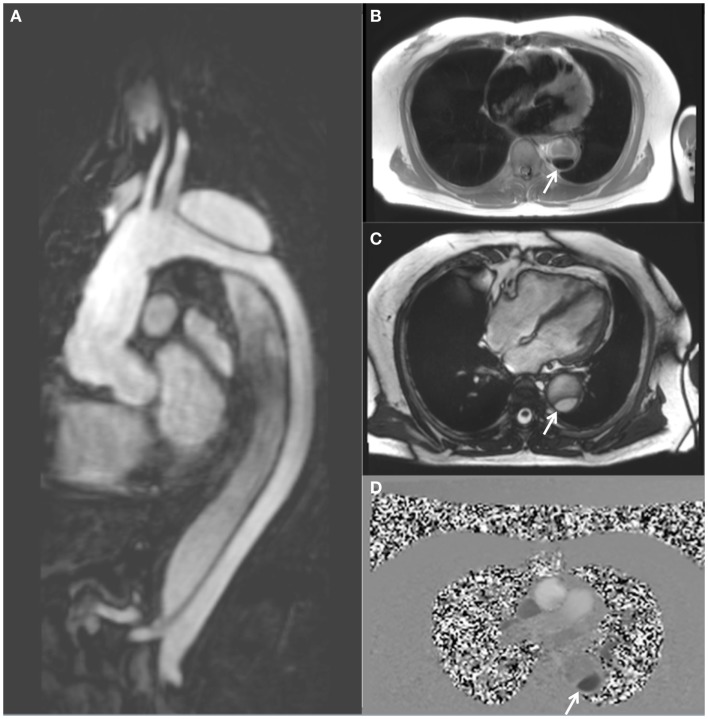
**MRI follow-up of a 63-year-old male with chronic descending thoracic aortic dissection**. The patient had undergone surgical replacement of the ascending aorta for type A aortic dissection 4 years earlier. **(A)** Sagittal gadolinium-contrast-enhanced MRA (magnetic resonance angiography) view; **(B)** axial black blood view of the proximal descending thoracic aorta; **(C)** axial true FISP (steady state-free precession) cine view; and **(D)** axial phase-contrast view, showing flow patterns in the true and false lumens of the descending aorta. The true lumen is indicated by the white arrow (Courtesy: Cardiovascular Magnetic Resonance, Sydney, Australia).

The most commonly used modalities are computed tomography (CT) and echocardiography, however magnetic resonance imaging (MRI) has recently emerged as a comprehensive, non-ionizing imaging tool well suited to serial measurements in this group of patients (Figure 1) (13). In the acute setting of aortic dissection, the most suitable imaging modality has been extensively examined, given the multiple critical clinical factors that affect time to diagnosis and appropriate management (2, 14, 15). Here, the utility of imaging modalities is limited by availability, time to acquire diagnostic imaging, cost, degree of invasiveness, the need for intravenous contrast, radiation exposure, and ease of intra-operative access. The availability of computed tomography angiography (CTA), together with the accurate visualization of the aortic root using trans-esophageal echocardiography (TEE), form the standard work-up in the majority of centers and offers a good combination of whole aortic coverage, characterization of dissection severity, and timely imaging (6). In the chronic setting, options for follow-up imaging are less constrained by the need for rapid image acquisition.

This review aims to compare the clinical utility of alternative imaging modalities for the follow-up of chronic aortic dissection by means of systematic review of the current literature, with emphasis upon the advantages and disadvantages of MRI in this setting, and discuss the future role of MRI in assessment of chronic aortic dissection.

## MRI and Chronic Aortic Dissection

We performed a systematic review of the current literature using pre-existing guidelines (16), to ascertain whether in patients with chronic aortic dissection, there is benefit from MRI-based follow-up when compared to other imaging modalities. The Cochrane Central Register of Controlled Trials, MEDLINE, and EMBASE were searched using the terms [(chronic dissection) and (aort*) and (MRI OR magnetic resonance imag*)]. Exclusion criteria were existing topic reviews, studies without a direct comparison of MRI and another imaging modality, and studies not in English. Case reports and conference abstracts were included due to a predicted paucity of available prospective trials.

**Figure 2:**
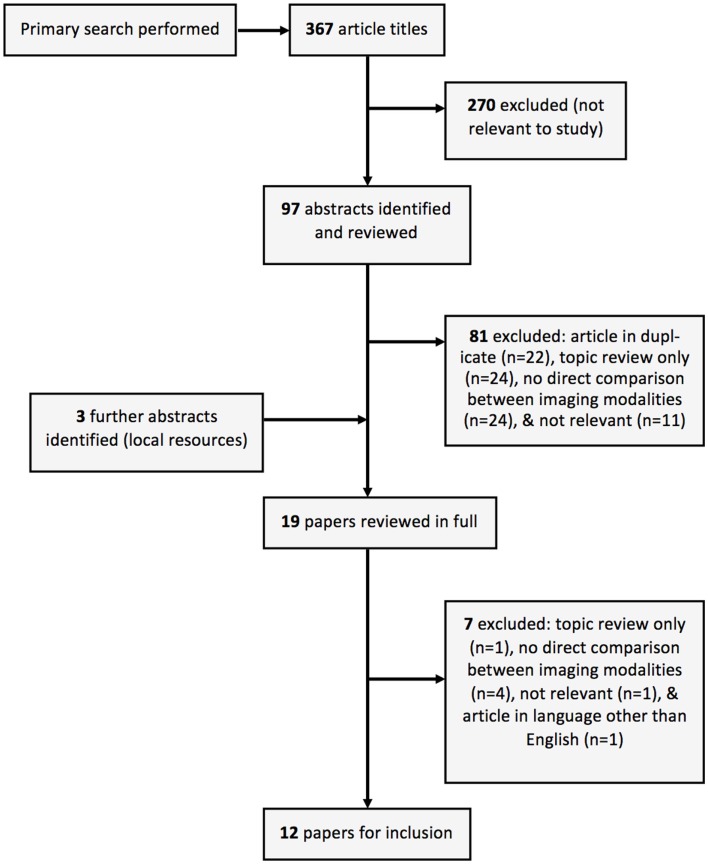
**Illustrated summary of study collection and inclusion**.

Twelve studies were included for this review from the literature selection process (summarized in Figure 2). No prospective studies were identified where patients with chronic dissection were randomized to follow-up with MRI or follow-up with another imaging modality. The included studies compared MRI and at least one other imaging modality, namely CT (17–21), TEE (22–26), traditional aortography (21, 27), or intravascular ultrasound (IVUS) (26, 28), in the follow-up of individual patients with heterogeneity in both MRI techniques utilized and reported radiological findings (Table 1). Additionally, the majority of included studies (10 of 12) were published in the year 2000 and earlier, when MRI technology was vastly different to today. Pertinent aspects of the included studies, related to the era of MRI use, are discussed below.

**Table 1:** **Patient and MRI data from the included studies**.

Reference	MRI patients, *n*	MRI tesla	MRI technique	Comparison imaging modality	Pathology
Clough et al. (17)	12	3.0	Breath-hold or respiratory-gated, ECG-triggered 3D SSFP	CT	Medically managed type B dissection
Bijnens et al. (18)	44	–	Not described	CT	Descending aortic dissection
Di Cesare et al. (22)	29	1.5	T1 spin-echo, cardiac-gated, and MRA	TEE	Descending aortic dissection following surgery for type A dissection
Maspes et al. (27)	2	1.5	Breath-hold 3D MRA	Aortography	Chronic aortic dissection
Cecconi et al. (23)	42	1.5	T1 spin-echo with ECG-gating ± gradient-echo	TEE	Descending aortic dissection following surgery for type A dissection
Masani et al. (24)	14	0.5	ECG and respiratory-gated T1 echo images	TEE	Descending aortic dissection following surgery for type A dissection
Yamada et al. (29)	7	1.5	Spin-echo	IVUS	Chronic aortic dissection
Deutsch et al. (25)	25	1.5	ECG-gated spin-echo or gradient-echo	TEE	Chronic aortic dissection
Williams et al. (26)	27	1.5	T1 spin-echo	IVUS and TEE	Aortic dissection
Rofsky et al. (19)	24	0.5	ECG-gated spin-echo ± gradient-echo FAME	CT	Descending aortic dissection following surgery for type A dissection
Grenier et al. (20)	17	0.5	Spin-echo ± ECG-gating	CT	Medically managed type B dissection
Pernes et al. (21)	29	0.5	Spin-echo ± ECG-gating	CT and aortography	Chronic aortic dissection

*MRI, magnetic resonance imaging; ECG, electrocardiography; 3D, three-dimensional; SSFP, steady state free precession; MRA, magnetic resonance angiography; FAME, fast acquisition with multiple excitation; CT, computed tomography; TEE, trans-esophageal echocardiography; IVUS, intravascular ultrasound*.

### The early application of MRI in chronic aortic dissection in 1980–2000

In addition to adequate assessment of FL patency, degree of thrombosis, and aortic diameter, MRI is capable of quantifying potentially prognostic hemodynamic parameters such as complex flow patterns, localized wall shear stress, valvular function, and pulse wave velocity. MRI technology is rapidly advancing, and recent improvements in gradient technology, radiofrequency coils, parallel imaging, pulse sequence design, and post-processing have greatly improved image quality. Despite the relatively rudimentary nature of early (from greater than 10 years ago) MRI studies utilizing low-field strengths (0.5–1.0 T) to assess chronic aortic dissection, results are favorable. Compared with CTA, the assessment of FL partial thrombosis was shown to be comparable (20, 30). Using spin-echo (SE) “black blood” techniques, the near absence of signal from blood flowing at a normal velocity affords a natural contrast that highlights flowing versus static blood in the aorta, in comparison to CTA which requires exogenous contrast to accurately define fluid compartments (20). Clinically useful assessment of FL patency was also demonstrated in early studies using phase-contrast (PC) gradient-echo (GRE) sequences to differentiate thrombus from slow flow (31).

### Current MRI technology

While these early studies highlighted the potential of MRI in imaging aortic dissection, current MRI scanners possess far superior technology and the ability to assess additional dynamic aspects of blood-flow and the aortic wall environment. This key potential advantage that MRI affords over other imaging modalities offers the prospect of developing new prognostic indicators that move beyond the simple measurement of vessel dimensions alone (32).

A typical MRI protocol for imaging of the aorta will include GRE and black blood-weighted images for anatomic definition (Figure 1B). The addition of steady state-free precession cine imaging (true FISP) affords a high blood/tissue contrast without the need for intravenous contrast administration (Figure 1C). Gadolinium-contrast-enhanced magnetic resonance angiography (MRA) (Figure 1A) provides high resolution three-dimensional (3D) data, similar to CT. However uncommon, gadolinium toxicity can nonetheless occur when such intravenous contrast is used, especially in those with pre-existing renal impairment (33).

The ability to assess and quantify blood-flow movement within the aorta is gained when PC imaging is utilized (Figure 1D). The ability to more reliably assess FL thrombosis (compared with CT) is an advantage of MRI in this context as FL thrombosis has been shown to be associated with reduced aortic expansion rates (7, 10, 34). Amano et al. reported their series of 16 chronic thoracic aortic dissection patients who underwent MRI at 1.5 T utilizing cardiac-gating, respiratory compensation, and fat suppression acquisition techniques together with 3D PC imaging of flow patterns (9, 35). They demonstrated that time-resolved 3D MRI may be used to assess the presence of blood-flow within the FL which has previously been shown to be prognostically significant (35, 36). In a group of 70 patients undergoing MRI (1.5 T) following surgically repaired type A aortic dissection, Almeida et al. demonstrated that the initial dimension of the descending thoracic aorta and the non-invasive pulse pressure to be independent predictors of late progression to aneurysm (37). Computational 3D models of descending thoracic aortic dissection have previously demonstrated a positive correlation between wall shear stress and disease progression (38).

### MRI as a problem-solving tool in chronic aortic dissection

An important application of MRI is in the identification of a number of important post-operative conditions. For example, the use of contrast-enhanced 3D MRA can distinguish aortic pseudoaneurysm from periprosthetic hematoma via identified areas of high signal intensity that are indicative of peri-graft flow (39). Furthermore, contrast-enhanced “breath-hold” MRA has been shown to be superior to “black blood” MRI for the assessment of intimal flaps and in assessing aortic branch vessel involvement (40). This is attributed to both the higher spatial resolution and the delineation of a hypointense intimal flap surrounded by contrast-enhanced bright blood (40). This benefit becomes particularly useful when classifying the dissection according to intimal tear location (41). A major advantage of MRI is the existence of multiple processing techniques that all provide specific and unique information. This permits a “problem-based” approach, where a number of different MRI sequences are used to characterize the features of the dissected aorta (17–21, 40).

Blood-flow quantification using PC or velocity mapping can demonstrate bidirectional flow within a FL; such turbulent flow may induce aortic wall shear stress which may be associated with elevated risk of aneurysmal dilatation or tear (22–26, 42). Pressure-dependent movement of a dissection membrane and consequent branch obstruction also indicates that morphologic assessment alone may be insufficient (21, 27, 42). Additionally, MRI affords the ability to accurately assess blood-flow at low velocities and hence differentiate this from FL thrombosis (17).

## Alternative Imaging Techniques

### Echocardiography

Echocardiography is generally tolerated well by patients and does not require use of contrast or ionizing radiation. It is also widely available and may be performed at the bedside. It can assess FL flow, FL thrombosis, and FL communication with the true lumen (TL); all significant prognostic markers (26, 28, 34, 43). Assessment of flow using echocardiography is optimal owing to high temporal resolution and is able to quantitate maximum flow velocity. However, the quantitative assessment of flow is limited due to the angular dependence of Doppler measurements, which makes this a highly operator-dependent modality. The recent addition of a 3D component to TEE has helped better quantify entry tear site and size (44). Early reports comparing it with MRI in the setting of chronic aortic dissection showed both modalities to be useful in follow-up, even when limited by inferior technology compared with more modern techniques (24, 25). In several studies examining patients following surgery for acute type A dissection, no difference between MRI and TEE was shown regarding the assessment of persistence and extent of aortic dissection (23). FL flow was better assessed with TEE in some cases, however, slow FL flow was unreliably detected by MRI sequences at this stage of technology development (23). A major limitation of echocardiography is the difficulty in viewing all sections of the thoracic aorta (23–25). MRI, as a cross-sectional modality, gives excellent coverage of the aorta throughout its course. Furthermore, MRI has shown lower inter-observer variability (compared with echocardiography) for aortic diameter measurement, a critical prognostic indicator (45).

In a study in 2000 by Di Cesare et al., 29 patients who had undergone surgery for type A dissection all underwent follow-up imaging with TEE, conventional MRI, and contrast-enhanced 3D breath-hold MRA (22). Imaging follow-up time ranged from 1 to 110 months post-operatively. A high correlation co-efficient was observed for diameter of the descending aorta in all three imaging types, however, TEE showed greater inter-observer variability of measurements made at the distal surgical anastomosis (22). Contrast-enhanced MRA was the most reliable in detection of FL flow; MRI was considered the modality of choice for the follow-up of surgically treated patients with persisting distal aortic dissection (22).

Although TEE can provide useful prognostic information in the acute setting, current recommendations suggest MRI or CT to be more useful for long-term follow-up (5, 43). Given its comparatively low cost, favorable temporal resolution, and bedside utility, previous recommendations have included TEE as a first line follow-up imaging modality in chronic dissection (23), however, this may not be entirely appropriate when measurement of absolute aortic dimensions are of such high importance.

### Computed tomography

Computed tomography angiography has essentially replaced conventional angiography in the assessment of aortic disease secondary to the reduced associated morbidity, lack of invasiveness, and lower cost associated with this modality. CTA is additionally readily available in the vast majority of clinical centers, and has a high reproducibility and low intra- and inter-operator variability (46). Its drawbacks include the need for iodinated contrast and the exposure to radiation. For patients greater than 60 years of age with normal renal function, the potential negative effects of such radiation exposure may be negligible when compared with the risks of their aortic disease (2), however the increasing recognition of multiple types of genetically defined aortopathies means that for younger patients requiring long-term monitoring, radiation dose may be an important consideration. New advances in reconstruction algorithms, prospective gating, and detector efficiency have permitted large reductions in radiation dose. Although not specific to chronic aortic dissection, this has been demonstrated in several recent reports, where “low radiation” CT assessment of aortic coarctation (47), endoleak (48), and the ascending aorta (49) has been achieved.

The time to complete a CTA examination is relatively short (4–20 s) and the use of electrocardiographic (ECG) gating techniques allows reliable artifact-free imaging of the aortic root and coronary arteries when necessary (5, 50, 51). CTA can show superior visualization of vessel calcification (compared with echocardiography or MRI where it is often observed as artifact or “signal drop-out”) and extremely precise aortic lumen diameter measurements. In the context of endovascular aortic stent-grafts and some mechanical heart valves, CT is deemed as the imaging modality of choice (4). MRI may become feasible in this subset of patients, however, inadequate visualization of stent struts and incompatibility with stainless steel implants remains at present (52). The presence of stainless steel wires used for operative sternal closure additionally inhibits image acquisition due to artifact.

Despite improvements in modern multi-detector CT technology, limited temporal resolution can prevent reliable resolution of rapidly moving structures such as an intimal flap or native valve leaflets (53). Ganten et al. have shown the potential for ECG-gated CTA imaging in chronic aortic dissection patients, particularly regarding determination of vessel distensibility (51). Thirty-two patients with conservatively treated type B dissection showed a reduction in aortic distensibility as measured by CTA (versus healthy age-matched controls), which may be a predisposing factor for dissection or a part of the vascular remodeling process following dissection (51). Such information may prove prognostically useful regarding the progression of aneurysmal disease. The feasibility of other novel CTA measures may also have potential as prognostic markers in chronic aortic dissection, for example, aortic displacement (a potential contributor to vessel wall shear stress) during the cardiac cycle (54), or four-dimensional (4D) CTA to assess aortic pulsatility (50).

### Other imaging modalities

As the traditional gold standard (2), aortography has been superseded by the less invasive approaches. Its high specificity and sensitivity are countered by risk of further iatrogenic aortic dissection and need for intravenous iodinated contrast (27, 55). Its use in the chronic dissection setting is difficult to justify given other available modalities. IVUS has similarly been reported to have both high sensitivity and specificity, however, is similarly compromised by its invasiveness (2, 29). The use of positron emission tomography (PET) has also been reported; in the acute setting areas of elevated metabolic activity at freshly disrupted segments of aortic wall show increased uptake of radionuclide tracer (12). In asymptomatic patients with chronic aortic dissection, however, no noticeable uptake is detected, hindering its use as a prognostic indicator in this setting (12). Furthermore, PET has limited spatial resolution, where precise delineation of fine structural detail is not always possible.

## Current Options of Advanced MRI in Chronic Dissection

### Computational fluid dynamics

The integration of computational fluid dynamics (CFD) as an adjunctive assessor of prognosis in chronic aortic dissection has been explored in several studies (56–58). Such approaches utilize computer-based algorithms involving Newtonian fluid flow and complement MRI to provide specific hemodynamic parameters. A case study from Karmonik et al. of a 45-year-old male with chronic aortic dissection where dual phase MRA and two-dimensional (2D) PC results were compared with CFD studies demonstrated that such simulation could quantify changes in both total pressure and wall shear stress during follow-up using patient-derived data (56). Karmonik’s group have additionally compared such changes with that which occurs in the healthy aorta, albeit with a small case series (*n* = 2) (58). When using MRI at 1.5 T and CFD, both the ascending aorta and TL diameter increased by a factor of 1.36 times in the chronic dissection patient compared with the patient with a healthy aorta. Abnormal wall shear stress values (considerably lower in the healthy aorta) were attributed to aneurysmal dilatation, rather than simply an increase in FL pressure or increase in the intra-arterial pressure gradient (58). As is noted in these studies, however, CFD simulations describe fixed mechanical forces and effects and do not take into account the multiple biological factors that exist in a native vessel.

### 4D flow MRI

**Figure 3:**
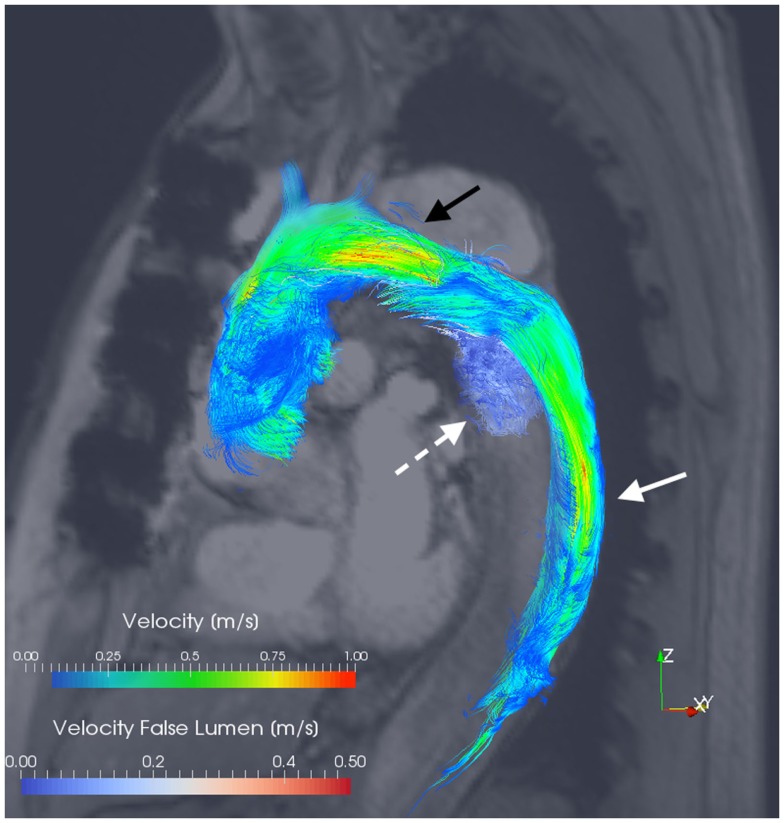
**3D velocity-encoded (4D) view with velocity streamlines of the thoracic aorta of the patient presented in Figure 1**. Blood-flow vectors passing through the true lumen (*solid white arrow*) and the false lumen (*dotted white arrow*) have been isolated; the reconstructed outline of the entire thoracic aorta is shown. Notably, flow acceleration is observed within the true lumen at the aortic arch (*black arrow*). Courtesy by Dr. F. Callaghan, Sydney Translational Imaging Laboratory, The Charles Perkins Centre, and The University of Sydney, Sydney, Australia.

Hemodynamic quantification appears to be the major advantage of MRI over other imaging modalities in chronic aortic dissection. Encoding of all three spatial directions of a volumetric data set utilizing 3D velocity-encoded cine MRI relative to the cardiac cycle, is commonly referred to as 4D flow MRI (59). The velocity and direction of aortic blood-flow can be represented as a “streamline” image (Figure 3). A reported MRI at 1.5 T of a single patient with chronic dissection from Müller-Eschner et al. with the use of velocity-colored “streamlines” of blood-flow showed acceleration of flow entering the FL through the primary entry tear (59). This evident vortical flow in the FL may be a further contributing factor to progressive expansion of the FL and aneurysmal dilatation. Maj et al. have similarly published hemodynamic information acquired using time-resolved, contrast-enhanced MRA (60). They show that with a sufficient blood velocity difference between the FL and TL, it is possible to achieve separate contrast enhancement of the dissected luminal channels (60).

Clough et al. have shown excellent accuracy of 4D PC MRI in velocity assessment (when compared with the MRI gold standard of 2D PC MRI) (17). In their series of 12 patients, the utilization of a 4D PC sequence demonstrated a correlation between the rate of rotation of helical blood-flow and the rate of aortic expansion in the FL (17). Francois et al. have similarly demonstrated the feasibility of integration of 4D flow techniques using MRI at 3 T, where the entire thoracic aorta can be imaged in a single acquisition without significant additional scan time (32). Their computation of blood-flow streamlines has allowed the observation of such helicity and vortical blood-flow as well as significantly more retrograde flow in the FL compared with the TL (32). Such approaches raise the potential to derive secondary biomarkers or wall shear stress forces from acquired 4D fields as clinical prognosticators (32, 61). The significance of such observations upon aortic disease progression, risk of future acute events, and ultimately patient mortality and morbidity is yet to be determined.

## Conclusion

In the setting of chronic aortic dissection, the ideal imaging modality for use in follow-up has high sensitivity and specificity, is non-invasive, and can accurately identify not only aortic dimensions but also progressive changes in relative flow between true and FLs. MRI can achieve these objectives, as well as the assessment of significant prognostic indicators such as FL thrombosis. CT and echocardiography remain the two most widely used alternatives, given their easy access, rapid acquisition, and non-invasiveness. There is a paucity in the current literature comparing these imaging modalities in the context of chronic aortic disease. In patients requiring multiple studies, the risks of repeated contrast/radiation exposure (especially in the young) and inter-observer variability in assessed aortic dimensions remain a concern. New techniques in MRI include more accurate hemodynamic assessment and the use of 4D imaging to demonstrate blood-flow and potential wall shear stress. However, the relevance of such techniques to long-term prognosis warrants further prospective investigation. Given its advantages, and when available, MRI is suggested as a suitable imaging modality in the follow-up of chronic aortic dissection.

## Conflict of Interest Statement

Cardiovascular Magnetic Resonance Sydney receives sponsorship funding from Siemens Ltd. (Australia). There was no involvement from Siemens in the development or preparation of this manuscript.

## Author’s Note

This manuscript was presented in poster format at the 2014 Australian and New Zealand Society of Cardiac and Thoracic Surgeons Annual Scientific Meeting (9–12 November, Gold Coast, Australia).

## Abbreviations

2D, two-dimensional; 3D, three-dimensional; 4D, four-dimensional; BAV, bicuspid aortic valve; CFD, computational fluid dynamics; CT, computed tomography; CTA, computed tomography angiography; ECG, electrocardiography; FL, false lumen; GRE, gradient-echo; IRAD, international registry of acute aortic dissection; IVUS, intravascular ultrasound; MFS, Marfan syndrome; MRA, magnetic resonance angiography; MRI, magnetic resonance imaging; PC, phase-contrast; PET, positron emission tomography; SE, spin-echo; T, Tesla; TAAD, thoracic aortic aneurysm and dissection syndrome; TL, true lumen; TEE, trans-esophageal echocardiography.
